# A Potential Functional Food-Based Neuroprotective Strategy Using Mulberry Leaf Extract and Trolox Against H_2_O_2_-Induced Oxidative Stress in SH-SY5Y Cells

**DOI:** 10.3390/foods15111974

**Published:** 2026-06-02

**Authors:** Nootchanat Mairuae, Jenjiralai Phanphak, Natechanok Thipboonchoo, Piyawan Wilaisit, Pornpimon Butsamongkon, Yutthana Chotritthirong, Sasalux Kaewbutra, Chanakarn Loiklung, Nut Palachai

**Affiliations:** 1 Faculty of Medicine, Mahasarakham University, Maha Sarakham 44000, Thailand; nootchanat.m@msu.ac.th (N.M.); jenjiralai.p@msu.ac.th (J.P.); natechanok.t@msu.ac.th (N.T.); piyawan.w@msu.ac.th (P.W.); ponpimon.b@msu.ac.th (P.B.); 2 Graduate School, Faculty of Pharmaceutical Sciences, Khon Kaen University, Khon Kaen 40002, Thailand; yutthana_ch@kkumail.com; 3 Department of Medical Science, Mahidol University, Amnatcharoen Campus, Amnat Charoen 37000, Thailand; sasalux.kae@mahidol.ac.th; 4 Unit of Water and Food Analysis, Department of Medical Science, Amnatcharoen Campus, Amnat Charoen 37000, Thailand; 5 Faculty of Veterinary Medicine, Khon Kaen University, Khon Kaen 40002, Thailand; chanakarn.l@kkumail.com

**Keywords:** mulberry leaf extract, vitamin E, Trolox, functional food, oxidative stress, neuroprotection, SH-SY5Y cells, neuron, antioxidant, apoptosis

## Abstract

This study investigated the synergistic potential of mulberry leaf extract and Trolox against hydrogen peroxide (H_2_O_2_)-induced oxidative stress in SH-SY5Y cells. Cells were treated with the combination for 24 h prior to exposure to 200 µM H_2_O_2_. Cell viability was assessed using the MTT assay, while oxidative status was evaluated through measurements of intracellular reactive oxygen species (ROS), malondialdehyde (MDA), and the activities of superoxide dismutase (SOD), catalase (CAT), and glutathione peroxidase (GSH-Px). Sirtuin 1 (SIRT1) and apoptosis-related proteins, including p53, cyclic AMP response element-binding protein (CREB), Bcl-2-associated X protein (Bax), and B-cell lymphoma 2 (Bcl-2), were determined by Western blot analysis. The combination treatment markedly upregulated SIRT1 expression, which was associated with increased CREB and Bcl-2 expression alongside reduced p53 and Bax levels. Increased SIRT1 expression was also accompanied by significant reductions in ROS and MDA levels and restoration of antioxidant enzyme activities. Collectively, these effects contributed to attenuation of oxidative stress and apoptosis, resulting in improved cell viability. These findings may support the formulation as a promising functional food-based strategy against oxidative stress-induced neuronal damage. However, these results are based on an in vitro SH-SY5Y cell model and represent preliminary evidence, warranting further in vivo and clinical studies to confirm their translational potential.

## 1. Introduction

Neurodegenerative conditions continue to place a significant burden on healthcare systems worldwide because their progression leads to persistent neurological decline and limited therapeutic options [1]. Among the multiple pathological mechanisms involved, oxidative stress is widely recognized as a key contributor to neuronal dysfunction and degeneration [2]. Excessive production and accumulation of ROS disrupt mitochondrial function, induce lipid peroxidation, and activate pro-apoptotic signaling pathways, ultimately resulting in progressive neuronal loss [3]. Mechanistically, hydrogen peroxide (H_2_O_2_) acts as a major mediator of oxidative damage due to its high stability and ability to readily penetrate cell membranes, where it rapidly generates highly reactive intracellular hydroxyl radicals that drive the core cascades of oxidative injury associated with neurodegenerative pathologies [4]. Therefore, strategies that effectively counteract oxidative stress are of considerable interest for neuroprotection.

Sirtuin 1 (SIRT1), a nicotinamide adenine dinucleotide (NAD^+^)-dependent deacetylase, has emerged as a critical determinant of neuronal survival and mitochondrial homeostasis [5]. SIRT1 protects against oxidative stress by suppressing ROS accumulation, enhancing endogenous antioxidant defenses, and modulating apoptotic signaling pathways [6,7]. Notably, its expression and activity are highly responsive to nutritional and metabolic status, suggesting that plant-derived functional ingredients may act as upstream modulators of neuronal resilience [8,9].

Plant-based bioactive compounds are increasingly recognized as safe and accessible approaches for modulating cellular regulatory pathways involved in neuronal function and disease susceptibility [10]. Mulberry (*Morus* spp.), traditionally used in Asian cuisine and herbal medicine, is rich in polyphenols with antioxidant and anti-apoptotic properties. Recent studies have shown that mulberry leaf extract enhances antioxidant defenses, reduces α-synuclein and amyloid-β accumulation, and alleviates tau-associated neuroinflammation and mitochondrial dysfunction through the restoration of redox balance and suppression of pro-inflammatory mediators, thereby improving neuronal survival [11,12]. Emerging evidence further suggests that mulberry-derived polyphenols may exert neuroprotective effects partly through modulation of SIRT1-related pathways associated with oxidative stress and mitochondrial function [13]. Collectively, these findings indicate that mulberry leaf-derived bioactive compounds may promote neuroprotection through multiple complementary mechanisms.

Despite these promising effects, mulberry leaf phytochemicals face practical limitations in functional food applications. Many bioactive constituents are unstable under physiological conditions, undergo extensive first-pass metabolism, and possess limited chemical stability, resulting in reduced bioavailability and the need for relatively high doses to achieve sufficient delivery to target tissues [14]. These challenges highlight the importance of combination strategies, where synergistic interactions may reduce the effective doses required to achieve potent biological effects.

Given these limitations, classical antioxidant agents represent a promising complement to mulberry leaf extracts. Trolox, a hydrophilic vitamin E analog, is well known for its potent free-radical scavenging capacity and its ability to prevent oxidative damage to cellular biomolecules [15]. Additionally, Trolox modulates apoptosis-related signaling and can cross the blood–brain barrier, enabling direct neuroprotective effects within the central nervous system [16]. From a translational perspective, combining a plant extract with an established and well-characterized compound such as Trolox may provide a more practical and accessible alternative to developing novel synthetic drugs, which often face high attrition rates, substantial financial burdens, and prolonged regulatory hurdles. When combined with mulberry leaf extract, Trolox may synergistically enhance neuroprotection through complementary molecular actions, including reinforcement of antioxidant defenses and suppression of apoptotic signaling, thereby improving cellular resilience. However, the cooperative effects of mulberry leaf extract and Trolox in neuronal cells remain insufficiently explored, particularly regarding their interactions with SIRT1-associated oxidative stress and apoptotic pathways.

Therefore, the present study aimed to investigate the neuroprotective effects of mulberry leaf extract combined with Trolox against H_2_O_2_-induced oxidative stress in SH-SY5Y neuroblastoma cells. We hypothesized that this combination would protect SH-SY5Y cells by upregulating SIRT1 protein expression and modulating oxidative stress parameters (ROS and MDA), enhancing endogenous antioxidant enzyme activities (SOD, CAT, and GSH-Px), and regulating mitochondrial apoptosis-related proteins (p53, CREB, Bax, and Bcl-2), ultimately leading to improved cell viability. The findings from this study may provide preliminary support for the development of functional food-based neuroprotective strategies aimed at mitigating oxidative stress-induced neuronal damage associated with neurodegenerative disorders.

## 2. Materials and Methods

### 2.1. Chemicals and Reagents

Trolox (6-hydroxy-2,5,7,8-tetramethylchromane-2-carboxylic acid; ≥98% purity) was purchased from Sigma-Aldrich (St. Louis, MO, USA). MTT reagent (3-(4,5-dimethylthiazol-2-yl)-2,5-diphenyltetrazolium bromide) and dimethyl sulfoxide (DMSO) were obtained from Merck KGaA (Darmstadt, Germany). Dulbecco’s Modified Eagle Medium (DMEM), fetal bovine serum (FBS), penicillin–streptomycin, non-essential amino acids (NEAA), and CM-H_2_DCFDA (5-(and-6)-chloromethyl-2′,7′-dichlorodihydrofluorescein diacetate) were purchased from Thermo Fisher Scientific (Waltham, MA, USA).

The human neuroblastoma cell line SH-SY5Y was obtained from the American Type Culture Collection (ATCC, Manassas, VA, USA) and maintained at the School of Biotechnology, Institute of Agricultural Technology, Suranaree University of Technology, Nakhon Ratchasima, Thailand.

Chemicals and reagents used for the assessment of oxidative stress and antioxidant enzyme activities included thiobarbituric acid (TBA; ≥98% purity), potassium permanganate (KMnO_4_; ≥95% purity), cytochrome C (≥95% purity), xanthine (≥99% purity), xanthine oxidase (from bovine milk; ≥95% purity), dithiothreitol (DTT; ≥99% purity), reduced glutathione (GSH; ≥98% purity), 5,5′-dithiobis-(2-nitrobenzoic acid) (DTNB; ≥98% purity), sodium dodecyl sulfate (SDS; ≥98.5% purity), acetic acid (CH_3_COOH; ≥99.7% purity), ethylenediaminetetraacetic acid (EDTA; ≥99% purity), sodium azide (NaN_3_; ≥99.5% purity), and 1,1,3,3-tetramethoxypropane (TMP; ≥96% purity), all obtained from MilliporeSigma (Burlington, MA, USA). Potassium dihydrogen phosphate (KH_2_PO_4_; ≥99.5% purity), hydrogen peroxide (H_2_O_2_; 30% *w*/*w*), and sulfuric acid (H_2_SO_4_; ≥95% purity) were purchased from Merck (Darmstadt, Germany).

For Western blotting, nitrocellulose membranes, SDS-polyacrylamide gels, and Tween-20 were supplied by Bio-Rad Laboratories (Hercules, CA, USA). Primary antibodies against SIRT1, p53, CREB, Bax, Bcl-2, and β-actin, along with horseradish peroxidase-conjugated secondary antibodies, were obtained from Cell Signaling Technology (Danvers, MA, USA). The Immobilon^®^ Forte Western HRP substrate was purchased from Merck KGaA.

### 2.2. Mulberry Leaf Material Handling and Extract Preparation

Mulberry leaves were supplied by the Queen Sirikit Department of Sericulture Center, Udon Thani Province, Thailand. To reduce natural variability, samples were collected from the same cultivation area and harvested at a consistent developmental stage. Fresh mulberry leaves were thoroughly washed with distilled water to remove surface contaminants and then air-dried at 25 °C under shaded conditions until a constant weight was achieved. The dried leaves were milled into a fine powder using a mechanical grinder.

Extraction was performed using 50% (*v*/*v*) ethanol as the solvent, which was selected due to its effectiveness in extracting a broad spectrum of polyphenolic compounds while maintaining compatibility with biological assays. In addition, aqueous ethanol is widely accepted for functional food research because of its relatively low toxicity and suitability for nutraceutical applications [17,18].

The powdered leaves were mixed with the extraction solvent at a solid-to-solvent ratio of 1:10 (*w*/*v*) and subjected to continuous agitation (150 rpm) at 25 °C for 24 h under light-protected conditions. The mixture was then filtered through Whatman No. 1 filter paper to remove insoluble residues. The filtrate was subsequently concentrated under reduced pressure using a rotary evaporator at 40 °C to remove ethanol and other volatile components. The remaining aqueous concentrate was lyophilized at −80 °C for 48 h to obtain dry mulberry leaf extract powder. The dried extract was stored at −20 °C in airtight containers protected from light until further use. Prior to cell culture experiments, the extract was freshly reconstituted in cell culture-grade DMSO, sterilized through a 0.22 µm membrane filter, and diluted to the desired working concentrations.

### 2.3. Identification and Quantification of Major Constituents in Mulberry Leaf Extract

Chemical profiling of the mulberry leaf extract was performed using reversed-phase HPLC with a Hypersil ODS column (4.0 × 250 mm, 5 µm; Agilent Technologies, Santa Clara, CA, USA) maintained at 25 °C. The mobile phase consisted of solvent A (0.05% phosphoric acid in water) and solvent B (acetonitrile), delivered at a flow rate of 0.60 mL/min. Gradient elution was initiated at 15% B (0.0 min), increased to 25% B at 3.0 min and maintained until 30 min, followed by an increase to 35% B at 35.0 min. The system was then re-equilibrated to 15% B at 40 min before the subsequent injection. Compound identification was performed by comparing retention times and UV spectral characteristics obtained from the DAD detector (200–800 nm) with those of authentic standards analyzed under identical chromatographic conditions [19].

Method validation was conducted in accordance with the International Council for Harmonisation (ICH) Q2(R2) guidelines [20]. Calibration curves were established over appropriate concentration ranges for each analyte, and all working solutions were prepared by serial dilution of 1 mg/mL stock solutions. For sample preparation, mulberry leaf extract (10 mg) was dissolved in methanol to obtain a 10 mg/mL stock solution and subsequently diluted with the mobile phase to the desired concentrations. Prior to analysis, all solutions were filtered through a 0.45 µm nylon membrane filter.

### 2.4. Cell Culture

SH-SY5Y human neuroblastoma cells were grown in Dulbecco’s Modified Eagle Medium (DMEM) supplemented with fetal bovine serum (10%) and antibiotics (penicillin 100 U/mL and streptomycin 100 µg/mL). Cultures were maintained under controlled conditions at 37 °C in a humidified incubator with 5% CO_2_ [21].

For experiments, cells were seeded at densities optimized for each assay. In 96-well plates used for cell viability and intracellular ROS measurements, cells were plated at 1 × 10^4^ cells per well. For biochemical and protein expression analyses, cells were cultured in 75 cm^2^ cell-culture flasks at approximately 1 × 10^6^ cells per flask. Cells were allowed to attach and recover overnight before any treatment.

### 2.5. Assessment of the Effective Combination and Interaction Between Mulberry Leaf Extract and Trolox

Initial screening experiments were conducted to determine concentration ranges of mulberry leaf extract and Trolox that did not compromise SH-SY5Y cell viability. Cells were exposed to increasing concentrations of each compound, and viability was assessed using the MTT assay. Concentrations that maintained at least 80% cell viability were considered suitable for combination testing.

To evaluate whether combined treatment enhanced protection against oxidative injury, concentration–response relationships for mulberry leaf extract and Trolox individual treatments, as well as their combinations, were established under H_2_O_2_-induced stress conditions. The concentrations producing approximately half-maximal protection for each agent were estimated and used as reference levels for combination analysis.

A series of combined treatments was then prepared by proportionally varying both agents around their reference concentrations, spanning lower and higher fractions of the estimated effective doses. To provide the necessary baseline data for synergy mathematical modeling, these individual concentrations and their respective combination pairs were tested concurrently. SH-SY5Y cells were pretreated with these single and combined treatments for 24 h prior to exposure to 200 µM H_2_O_2_ for an additional 24 h, and cell viability data were used to construct interaction matrices.

Quantitative evaluation of compound interactions was performed using the SynergyFinder platform (version 3.10.3). This analysis generated numerical interaction scores and visual interaction maps, enabling identification of concentration regions in which the combined treatment produced effects exceeding those expected from simple additivity.

To further substantiate the nature of the interaction, the Combination Index (CI) was calculated using the Chou–Talalay approach, comparing the doses required to achieve equivalent protective effects when administered alone or in combination. CI values below 1 were interpreted as evidence of synergistic interaction [22].

### 2.6. Experimental Design and Treatment Protocol

Experimental conditions were divided into four groups: control, vehicle, MLE–T1, and MLE–T2. The control group consisted of untreated cells serving as the baseline. The vehicle group received the same final concentration of DMSO, followed by H_2_O_2_ exposure to exclude solvent effects. Mulberry leaf extract and Trolox were freshly dissolved in cell culture-grade DMSO prior to dilution in culture medium. For the treatment groups, cells received combined mulberry leaf extract and Trolox at the selected ratios. To simulate a preventive strategy, a pre- and co-treatment approach was employed in which cells were incubated with the combinations and H_2_O_2_ at the concentrations and durations described earlier.

### 2.7. Cell Viability Assay

Cell viability was evaluated using the MTT assay [23]. At the completion of treatments, the culture medium was replaced with MTT-containing medium (0.5 mg/mL), and plates were incubated for 1 h at 37 °C. The reaction was stopped by removing the MTT solution, and the resulting formazan crystals were solubilized in DMSO.

Absorbance was recorded at 570 nm using a microplate reader (BioTek Instruments, Winooski, VT, USA). Viability values were normalized to the untreated control group. The percentage of cell viability was calculated using the following formula:

%Cell viability = [(A_Sample_ − A_Blank_)/(A_Control_ − A_Blank_)] × 100

where A_Sample_ represents the absorbance of treated cells, A_Control_ represents the absorbance of untreated control cells, and A_Blank_ represents the absorbance of blank wells without cells.

The cytoprotective efficacy was subsequently determined as the percentage inhibition of H_2_O_2_-induced cytotoxicity using the following formula:

%Inhibition = [(A_Sample_ − A_Toxin_)/(A_Control_ − A_Toxin_)] × 100

where A_Toxin_ represents the absorbance of cells treated with H_2_O_2_ alone.

### 2.8. Intracellular Reactive Oxygen Species Analysis

Changes in intracellular reactive oxygen species were monitored using the oxidation-sensitive fluorescent probe DCFH-DA. SH-SY5Y cells grown in 96-well plates were rinsed with phosphate-buffered saline and incubated with DCFH-DA at a final concentration of 10 µM for 30 min at 37 °C under light-protected conditions to allow intracellular de-esterification of the probe. Excess dye was removed by gentle washing before cells were subjected to oxidative challenge in the presence or absence of the designated treatments.

Fluorescence generated by oxidized dichlorofluorescein was detected using a microplate reader (BioTek Instruments) with excitation set at 488 nm and emission at 520 nm. Fluorescence values were corrected for background signal and expressed relative to the corresponding control group to reflect changes in intracellular ROS levels [24].

### 2.9. Lipid Peroxidation and Antioxidant Enzyme Analyses

MDA levels and antioxidant enzyme activities were determined using standardized biochemical assays based on previously validated protocols. Detailed assay protocols were described in our previous study [25]. Briefly, treated cells were homogenized in 0.1 M potassium phosphate buffer (pH 7.4) at a ratio of 10 mg sample to 50 µL buffer. Total protein concentration was determined at 280 nm using a NanoDrop™ 2000c spectrophotometer (Thermo Fisher Scientific, Wilmington, DE, USA) for sample normalization.

MDA levels were determined using the TBA reaction. Samples were mixed with SDS, TBA, and CH_3_COOH, heated at 95 °C for 60 min, and extracted with n-butanol:pyridine (15:1, *v*/*v*) prior to centrifugation, after which the absorbance of the upper layer was measured at 532 nm.

CAT activity was assessed based on the decomposition of H_2_O_2_ using 30 mM H_2_O_2_ in 50 mM phosphate buffer (pH 7.0), with the reaction terminated by 4 M H_2_SO_4_ and 5 mM KMnO_4_ before absorbance measurement at 490 nm.

SOD activity was evaluated using the xanthine–xanthine oxidase/cytochrome C system containing xanthine, phosphate buffer, EDTA, and cytochrome C, followed by absorbance measurement at 415 nm after the addition of xanthine oxidase.

GSH-Px activity was analyzed using a reaction mixture containing DTT, NaN_3_, GSH, and H_2_O_2_, followed by incubation at 25 °C for 10 min, DTNB color development, and absorbance measurement at 412 nm.

### 2.10. Western Blot Analysis

After treatments, SH-SY5Y cells were collected and lysed in Neuronal Protein Extraction Reagent (N-PER) containing protease inhibitor cocktail. Lysates were centrifuged at 10,000× *g* for 10 min at 4 °C, and the supernatant was retained for protein quantification using a NanoDrop 2000c spectrophotometer.

For electrophoresis, 60 µg of protein was mixed with SDS loading buffer, denatured at 95 °C for 5 min, and separated on 10% SDS–polyacrylamide gels using Tris–glycine–SDS running buffer under a constant voltage of 120 V for approximately 1 h. Proteins were then transferred to PVDF membranes using a semi-dry transfer system at 18 V for 1 h in Tris–glycine transfer buffer containing 10% methanol.

Membranes were incubated overnight at 4 °C with gentle rocking with primary antibodies: anti-SIRT1, anti-CREB, anti-p53, anti-Bax, and anti-Bcl-2 (1:1000). After three washes with 0.05% TBS-T, membranes were incubated with HRP-conjugated anti-rabbit IgG secondary antibody (1:2000) for 1 h at 25 °C. Protein signals were detected using Immobilon Forte Western HRP Substrate and captured on a ChemiDoc MP imaging system.

Band intensities were quantified with Image Lab software (version 6.0) and normalized to β-actin. Protein expression levels were expressed relative to the control group [26].

### 2.11. Statistical Analysis

All experiments were conducted in at least three independent replicates, and data are presented as mean ± standard error of the mean (SEM). The normality of the datasets was assessed using the Shapiro–Wilk test, and homogeneity of variances was evaluated with Levene’s test. Group comparisons were performed using one-way analysis of variance (ANOVA) followed by Tukey’s post hoc test for multiple comparisons. Statistical analyses were conducted with SPSS Statistics version 21.0 (IBM Corp., Armonk, NY, USA), and a *p*-value < 0.05 was considered statistically significant.

## 3. Results

### 3.1. HPLC-Based Identification and Quantification of Major Constituents in Mulberry Leaf Extract

**Figure 1:**
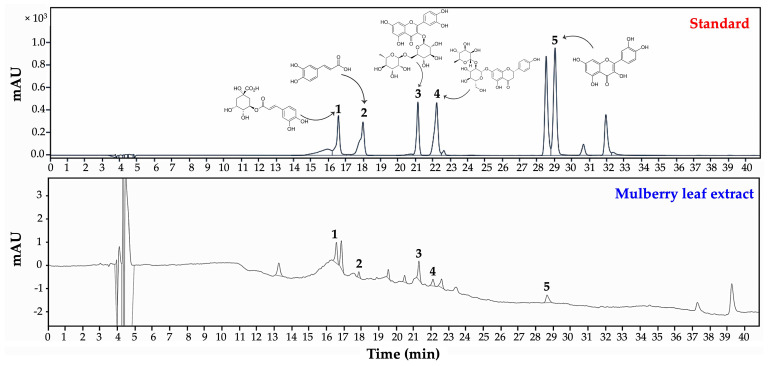
High-performance liquid chromatography (HPLC) chromatogram of major constituents identified in mulberry leaf extract.

The developed HPLC–DAD method provided satisfactory separation of the major phenolic and flavonoid constituents in mulberry leaf extract within 40 min. Representative chromatograms of the authentic standards and mulberry leaf extract are shown in Figure 1. Compound identification was performed by comparing retention times and UV spectral characteristics obtained from the DAD detector with those of the corresponding authentic standards. Peaks corresponding to chlorogenic acid (1), caffeic acid (2), rutin (3), naringin (4), and quercetin (5) were identified in the mulberry leaf extract based on comparison with authentic standards.

Method validation demonstrated excellent linearity for all analytes, with correlation coefficients (R^2^) exceeding 0.99. The method also showed acceptable precision, with intra- and inter-day %RSD values below 3%. Accuracy, assessed by recovery studies, ranged from 98% to 101%, confirming the reliability and reproducibility of the analytical method.

Quantitative HPLC analysis revealed that chlorogenic acid was the predominant phenolic compound in mulberry leaf extract (1.00 ± 0.01 mg/g extract), followed by rutin (0.45 ± 0.001 mg/g extract), quercetin (0.39 ± 0.01 mg/g extract), naringin (0.36 ± 0.001 mg/g extract), and caffeic acid (0.12 ± 0.002 mg/g extract). The concentration of each compound is summarized in Table 1.

### 3.2. Determination of Non-Toxic Concentrations of Mulberry Leaf Extract and Trolox

**Figure 2:**
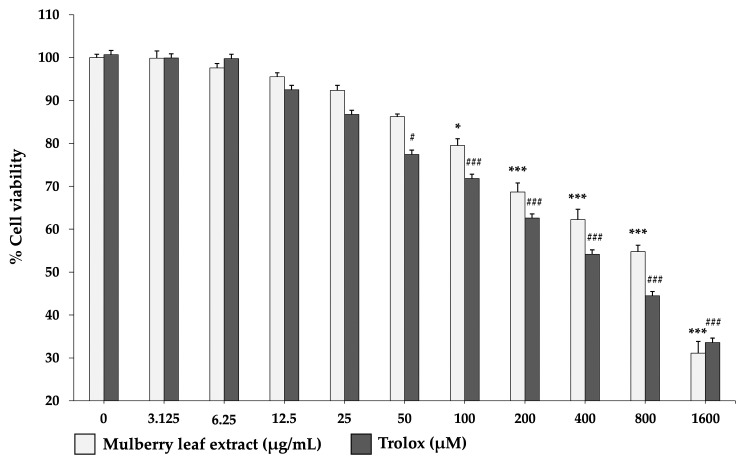
Effects of mulberry leaf extract and Trolox on the viability of SH-SY5Y neuronal cells. Cells were treated with increasing concentrations of MLE (0 to 1600 µg/mL) or Trolox (0 to 1600 µM) for 24 h, and cell viability was assessed using the MTT assay. Data are presented as mean ± SEM. For mulberry leaf extract, * *p* < 0.05 and *** *p* < 0.001 vs. control (0 µg/mL); for Trolox, ^#^ *p* < 0.05 and ^###^ *p* < 0.001 vs. control (0 µM).

The cytotoxicity of mulberry leaf extract and Trolox was first evaluated to establish safe concentrations for subsequent experiments. SH-SY5Y cells were exposed to increasing concentrations of each compound for 24 h, and cell viability was determined using the MTT assay. As shown in Figure 2, mulberry leaf extract did not induce significant cytotoxic effects at concentrations up to 50 µg/mL, with cell viability remaining comparable to the untreated control group. In contrast, higher concentrations produced a slight reduction in cell viability, suggesting the onset of dose-dependent toxicity. Similarly, Trolox treatment maintained cellular viability at concentrations up to 25 µM, whereas higher doses resulted in a further decrease in cell viability. These findings indicate that both agents are well tolerated within specific concentration ranges in SH-SY5Y cells.

Based on the viability profiles, concentrations that preserved greater than 80 percent cell survival were considered non-toxic and selected for further investigation. Therefore, mulberry leaf extract at concentrations up to 50 µg/mL and Trolox up to 25 µM were used in all subsequent experiments to ensure that any observed effects reflected neuroprotective activity rather than cytotoxicity.

### 3.3. Concentration-Dependent Cytoprotection and EC_50_ Estimation of Mulberry Leaf Extract and Trolox

**Figure 3:**
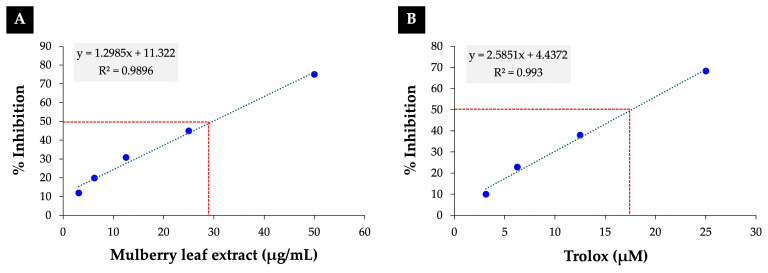
Concentration-dependent protective effects of (**A**) mulberry leaf extract and (**B**) Trolox against H_2_O_2_-induced cytotoxicity in SH-SY5Y cells. Cell viability was measured by the MTT assay. EC_50_ values were estimated from linear regression analysis of the concentration–response curves.

The protective effects of mulberry leaf extract and Trolox against H_2_O_2_-induced cytotoxicity were evaluated by pretreating SH-SY5Y cells with increasing concentrations of each compound prior to oxidative challenge. Cell viability was subsequently determined using the MTT assay. As shown in Figure 3, both mulberry leaf extract and Trolox improved cell survival in a concentration-dependent manner, indicating attenuation of H_2_O_2_-induced injury.

To estimate their protective potency, concentration–response data were analyzed. Because the selected concentration ranges fell within the linear dynamic portion of the sigmoidal dose–response curve and did not include definitive upper and lower asymptotic plateaus, linear regression modeling was employed as an appropriate approach for midpoint estimation. The EC_50_ values were calculated directly from the linear portion of the regression curves, yielding values of 29.79 µg/mL for mulberry leaf extract and 17.63 µM for Trolox. These estimated concentrations were subsequently used as reference levels for all combination experiments.

### 3.4. Synergistic Interaction Between Mulberry Leaf Extract and Trolox

**Figure 4:**
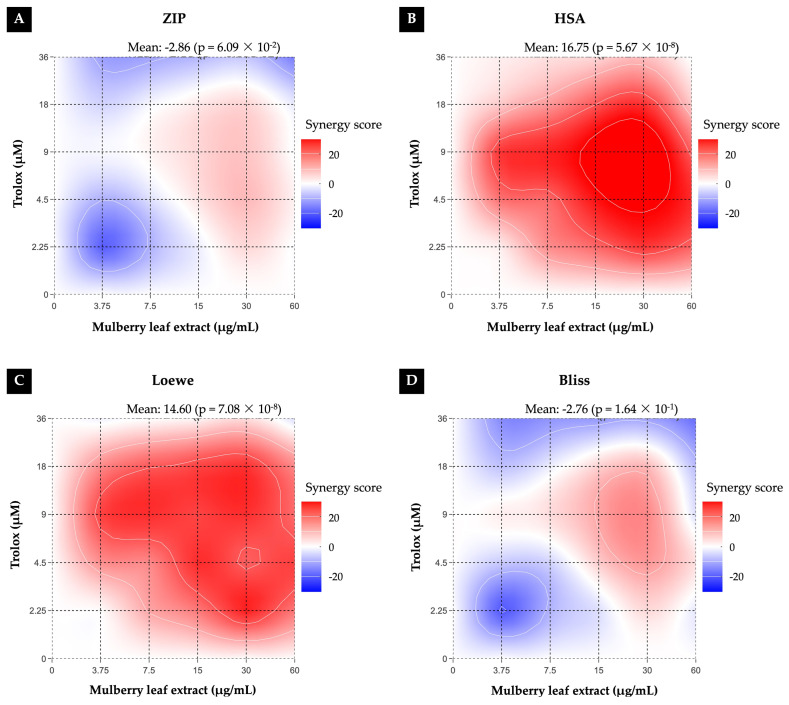
Synergy analysis of mulberry leaf extract combined with Trolox in H_2_O_2_-treated SH-SY5Y cells. Heatmaps show synergy scores calculated using (**A**) ZIP, (**B**) HSA, (**C**) Loewe, and (**D**) Bliss models. Positive scores indicate synergistic effects, values near zero indicate additive effects, and negative scores indicate antagonism. ZIP, Zero Interaction Potency; HSA, Highest Single Agent; Loewe, Loewe additivity; Bliss, Bliss independence.

To further determine whether the combined administration of mulberry leaf extract and Trolox enhanced cytoprotection beyond their individual effects, a matrix of concentration combinations was evaluated under H_2_O_2_-induced oxidative stress. Cell viability data were subjected to synergy analysis using multiple reference models, including Zero Interaction Potency (ZIP), Highest Single Agent (HSA), Loewe additivity, and Bliss independence. The resulting interaction landscapes are presented as heatmaps in Figure 4.

The HSA (Figure 4B) and Loewe (Figure 4C) models demonstrated predominantly positive interaction scores, with mean synergy scores of 16.75 and 14.6, respectively, indicating synergistic cytoprotective effects of the combined treatment. Higher synergy regions were mainly observed at moderate to high concentrations of both mulberry leaf extract and Trolox. In contrast, the ZIP (Figure 4A) and Bliss (Figure 4D) models produced mean synergy scores of −2.86 and −2.76, respectively, suggesting additive or slightly antagonistic interactions.

### 3.5. Combination Index Analysis and Selection of Optimal Dose Ratios for Subsequent Experiments

To further quantify the interaction between mulberry leaf extract and Trolox, combination effects were evaluated using the combination index (CI) method across different concentration ratios. The results are summarized in Table 2.

Among the tested combinations, 30 µg/mL mulberry leaf extract combined with 9 µM Trolox (1X:0.5X) and 30 µg/mL mulberry leaf extract combined with 18 µM Trolox (1X:1X) produced the lowest CI values, indicating the strongest synergistic cytoprotective effects. These findings were consistent with the synergy landscape shown in Figure 4, where moderate to higher concentrations of both agents yielded higher synergy scores. Based on these favorable interaction profiles, the two combinations were designated as MLE-T1 and MLE-T2, respectively, and selected for subsequent mechanistic studies.

### 3.6. Effects of Combined Treatment on Cell Viability Under Oxidative Stress

**Figure 5:**
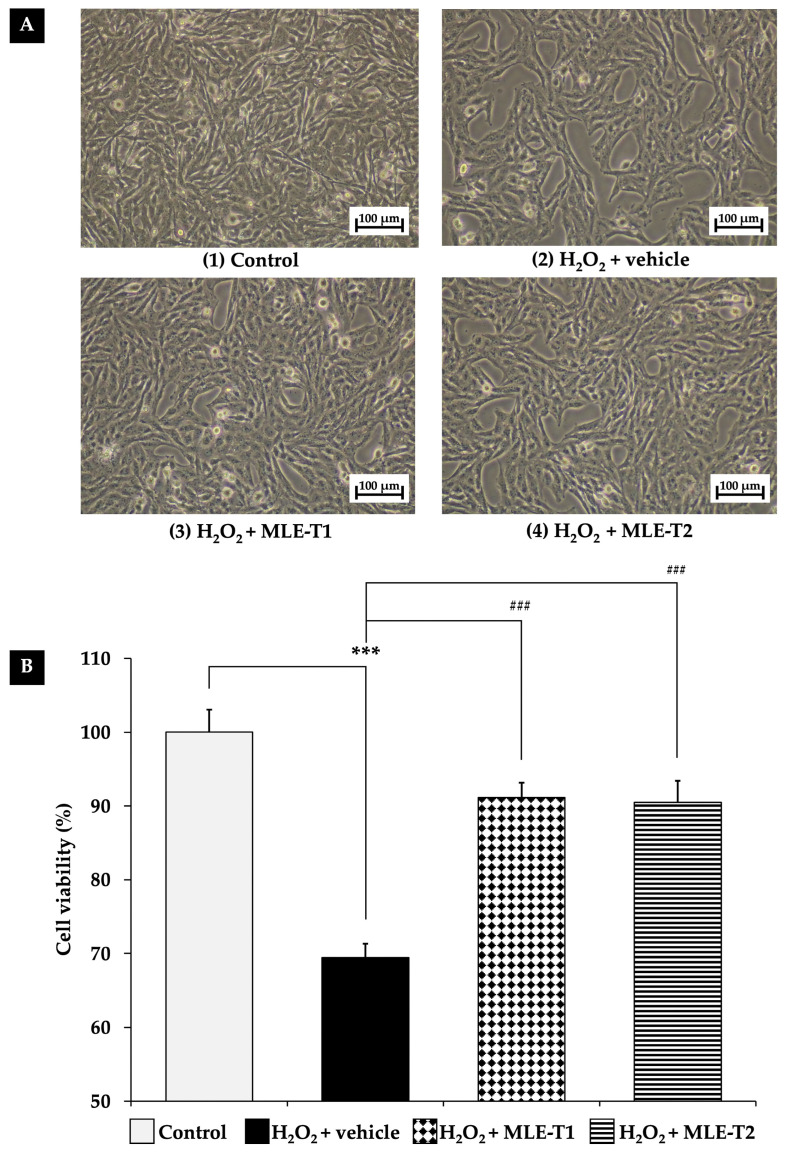
Effects of the selected mulberry leaf extract and Trolox combinations on SH-SY5Y cell viability under H_2_O_2_-induced oxidative stress. (**A**) Representative microscopic images showing cell morphology (10× magnification). (**B**) Cell viability determined by the MTT assay and expressed as percentage cell viability. Data are presented as mean ± SEM. *** *p* < 0.001 vs. control; ^###^ *p* < 0.001 vs. H_2_O_2_ plus vehicle. H_2_O_2_, hydrogen peroxide; MLE-T1, mulberry leaf extract 1X plus Trolox 0.5X; MLE-T2, mulberry leaf extract 1X plus Trolox 1X.

The protective effects of the selected combinations on H_2_O_2_-induced cytotoxicity were further evaluated by examining cellular viability. As shown in Figure 5A, untreated SH-SY5Y cells exhibited dense cellular distribution and normal attachment. In contrast, exposure to H_2_O_2_ visibly reduced cell density compared with the control group, indicating oxidative stress-induced cellular damage.

Treatment with the combined formulations partially preserved cellular distribution compared with the H_2_O_2_ plus vehicle group. Although some gaps between cells and cellular debris remained observable, cells treated with MLE-T1 and MLE-T2 appeared to exhibit higher cell density than the H_2_O_2_-treated group, suggesting partial protection against oxidative stress-induced cellular damage. Consistent with these observations, quantitative analysis using the MTT assay (Figure 5B) demonstrated that H_2_O_2_ exposure significantly decreased cell viability compared with the control group (*p* < 0.001). Treatment with both MLE-T1 and MLE-T2 significantly increased cell viability compared with the H_2_O_2_ plus vehicle group (*p* < 0.001), indicating protective effects of the combined treatments.

### 3.7. Effects of Combined Treatment on Oxidative Stress Markers and Antioxidant Enzyme Activities

To further clarify whether the observed improvements in cell viability were associated with modulation of oxidative stress, intracellular oxidative damage and antioxidant defense systems were evaluated by measuring MDA levels, ROS, and endogenous antioxidant enzyme activities.

**Figure 6:**
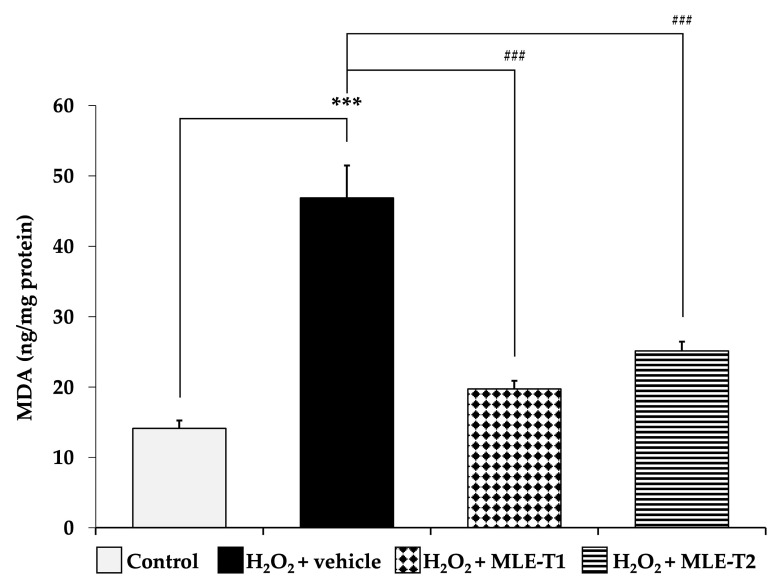
Effects of the selected mulberry leaf extract and Trolox combinations on lipid peroxidation (MDA) under H_2_O_2_-induced oxidative stress. Data are presented as mean ± SEM. *** *p* < 0.001 vs. control; ^###^ *p* < 0.001 vs. H_2_O_2_ plus vehicle. H_2_O_2_, hydrogen peroxide; MLE-T1, mulberry leaf extract 1X plus Trolox 0.5X; MLE-T2, mulberry leaf extract 1X plus Trolox 1X.

As shown in Figure 6, H_2_O_2_ plus vehicle significantly increased MDA levels compared with the control group (*p* < 0.001), indicating enhanced lipid peroxidation. Treatment with both MLE-T1 and MLE-T2 markedly reduced MDA levels relative to the H_2_O_2_-treated group (*p* < 0.001 for both).

**Figure 7:**
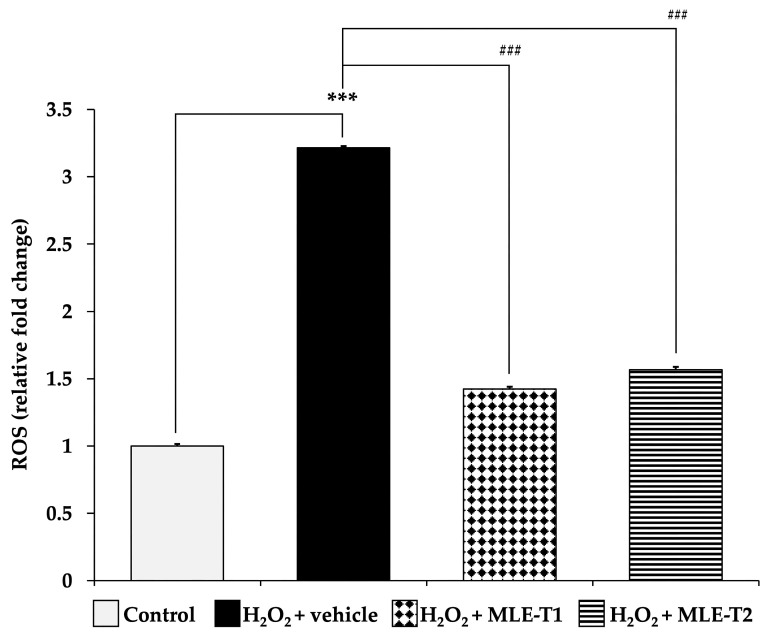
Effects of the selected mulberry leaf extract and Trolox combinations on intracellular ROS generation, measured using the DCFH-DA fluorescent probe, under H_2_O_2_-induced oxidative stress. Data are presented as mean ± SEM. *** *p* < 0.001 vs. control; ^###^ *p* < 0.001 vs. H_2_O_2_ plus vehicle. H_2_O_2_, hydrogen peroxide; MLE-T1, mulberry leaf extract 1X plus Trolox 0.5X; MLE-T2, mulberry leaf extract 1X plus Trolox 1X; ROS, reactive oxygen species.

Similarly, intracellular ROS production was markedly elevated following H_2_O_2_ exposure (Figure 7, *p* < 0.001 vs. control). Treatment with MLE-T1 and MLE-T2 significantly suppressed ROS accumulation compared with the H_2_O_2_ plus vehicle group (*p* < 0.001 for both).

**Figure 8:**
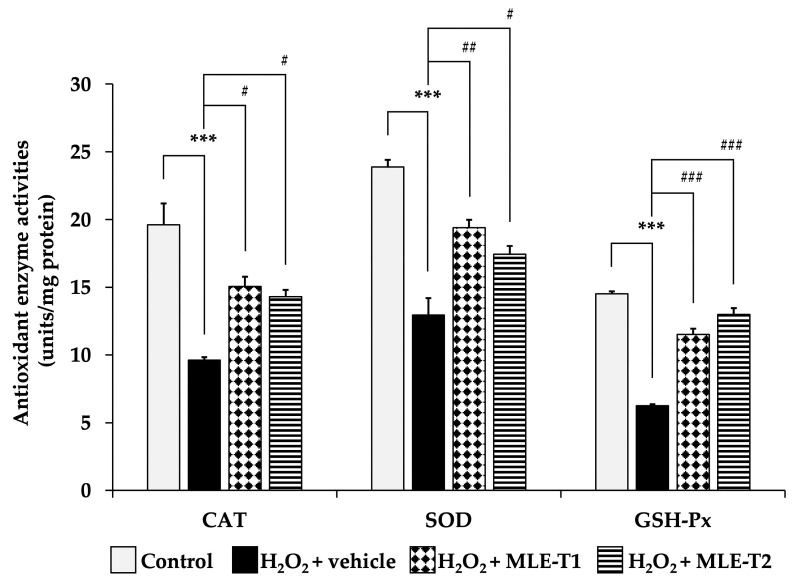
Effects of the selected mulberry leaf extract and Trolox combinations on antioxidant enzyme activities under H_2_O_2_-induced oxidative stress. Data are presented as mean ± SEM. *** *p* < 0.001 vs. control; ^#^ *p* < 0.05, ^##^ *p* < 0.01, and ^###^ *p* < 0.001 vs. H_2_O_2_ plus vehicle. H_2_O_2_, hydrogen peroxide; MLE-T1, mulberry leaf extract 1X plus Trolox 0.5X; MLE-T2, mulberry leaf extract 1X plus Trolox 1X. CAT, catalase; SOD, superoxide dismutase; GSH-Px, glutathione peroxidase.

Consistent with these findings, antioxidant enzyme activities were significantly impaired by oxidative challenge. As presented in Figure 8, H_2_O_2_ treatment decreased CAT, SOD, and GSH-Px activities compared with the control (*p* < 0.001 for all). Both combination treatments significantly restored CAT activity (*p* < 0.05 for both), increased SOD activity (*p* < 0.01 for MLE-T1 and *p* < 0.05 for MLE-T2), and markedly elevated GSH-Px activity (*p* < 0.001 for both).

### 3.8. Effects of Combined Treatment on SIRT1 Expression

To further investigate the molecular mechanisms underlying the observed antioxidant and cytoprotective effects, the expression of SIRT1, a key regulator of cellular stress resistance and survival signaling, was examined.

**Figure 9:**
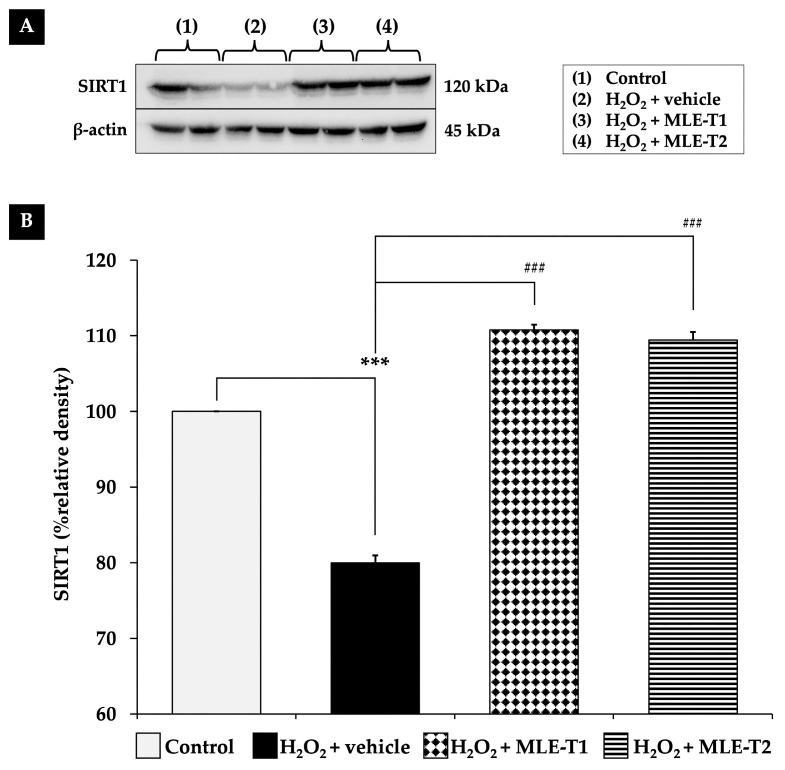
Effects of the selected mulberry leaf extract and Trolox combinations on sirtuin SIRT1 expression under H_2_O_2_-induced oxidative stress. (**A**) Representative Western blot images. (**B**) Quantitative analysis of SIRT1 expression relative to the control. Data are presented as mean ± SEM. *** *p* < 0.001 vs. control; ^###^ *p* < 0.001 vs. H_2_O_2_ plus vehicle. H_2_O_2_, hydrogen peroxide; MLE-T1, mulberry leaf extract 1X plus Trolox 0.5X; MLE-T2, mulberry leaf extract 1X plus Trolox 1X; SIRT1, sirtuin 1.

As shown in Figure 9, exposure to H_2_O_2_ plus vehicle significantly reduced SIRT1 expression compared with the control group (*p* < 0.001), indicating suppression of endogenous protective pathways under oxidative stress. In contrast, treatment with both MLE-T1 and MLE-T2 markedly increased SIRT1 expression relative to the H_2_O_2_-treated group (*p* < 0.001 for both). The restoration of SIRT1 levels was consistent with the improvements observed in cell viability and redox balance.

### 3.9. Effects of Combined Treatment on CREB and Bcl-2 Expression

Following the restoration of SIRT1 expression, the downstream survival-associated proteins CREB and Bcl-2 were evaluated to further clarify the molecular mechanisms contributing to cytoprotection.

**Figure 10:**
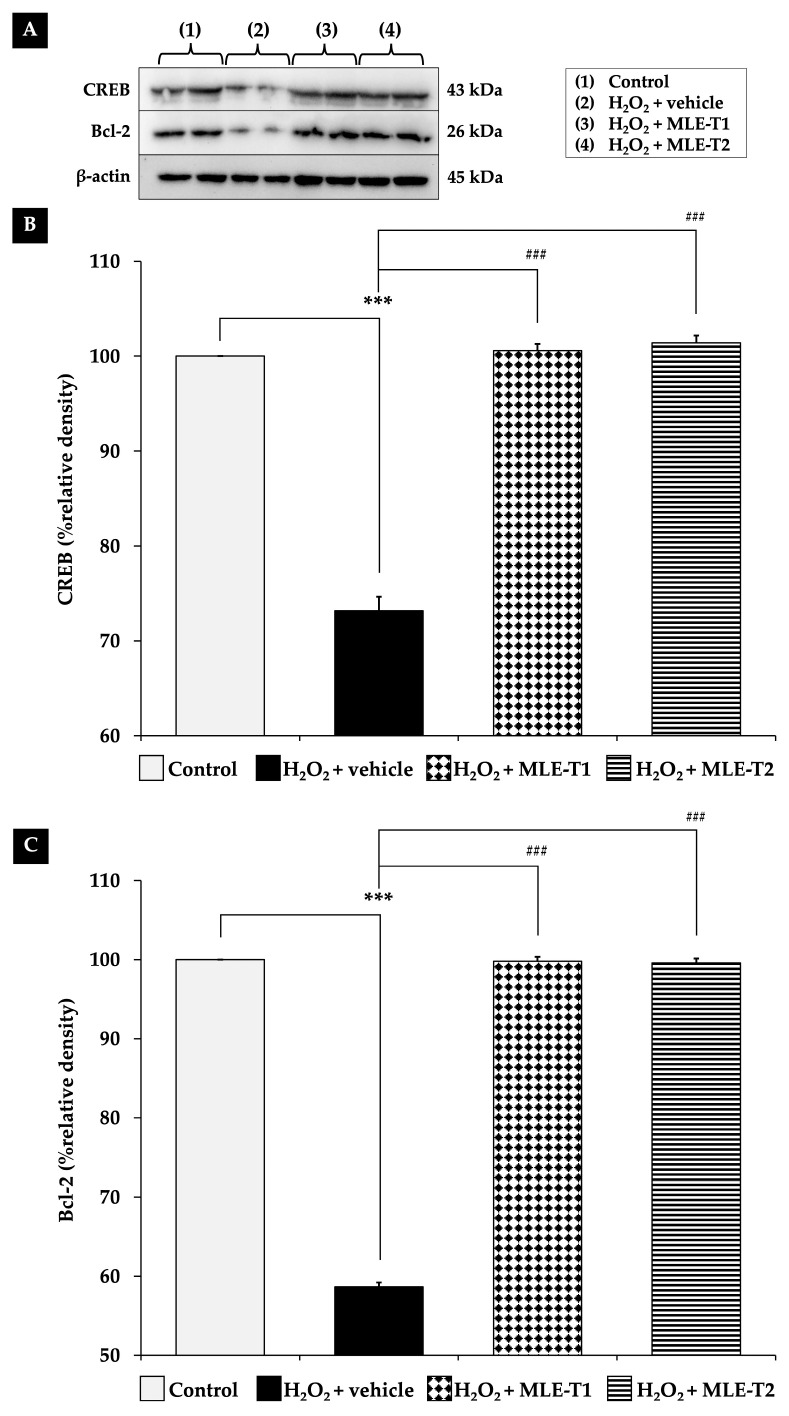
Effects of the selected mulberry leaf extract and Trolox combinations on CREB and Bcl-2 expression under H_2_O_2_-induced oxidative stress. (**A**) Representative Western blot images. (**B**) Quantitative analysis of CREB expression relative to the control. (**C**) Quantitative analysis of Bcl-2 expression relative to the control. Data are presented as mean ± SEM. *** *p* < 0.001 vs. control; ^###^ *p* < 0.001 vs. H_2_O_2_ plus vehicle. H_2_O_2_, hydrogen peroxide; MLE-T1, mulberry leaf extract 1X plus Trolox 0.5X; MLE-T2, mulberry leaf extract 1X plus Trolox 1X; CREB, cyclic AMP response element-binding protein; Bcl-2, B-cell lymphoma 2.

As shown in Figure 10, exposure to H_2_O_2_ plus vehicle significantly reduced the expression levels of both CREB and Bcl-2 compared with the control group (*p* < 0.001), indicating suppression of pro-survival signaling under oxidative stress conditions. Treatment with the selected combinations, MLE-T1 and MLE-T2, markedly increased the expression of CREB and Bcl-2 relative to the H_2_O_2_-treated group (*p* < 0.001 for all).

### 3.10. Effects of Combined Treatment on p53 and Bax Expression

**Figure 11:**
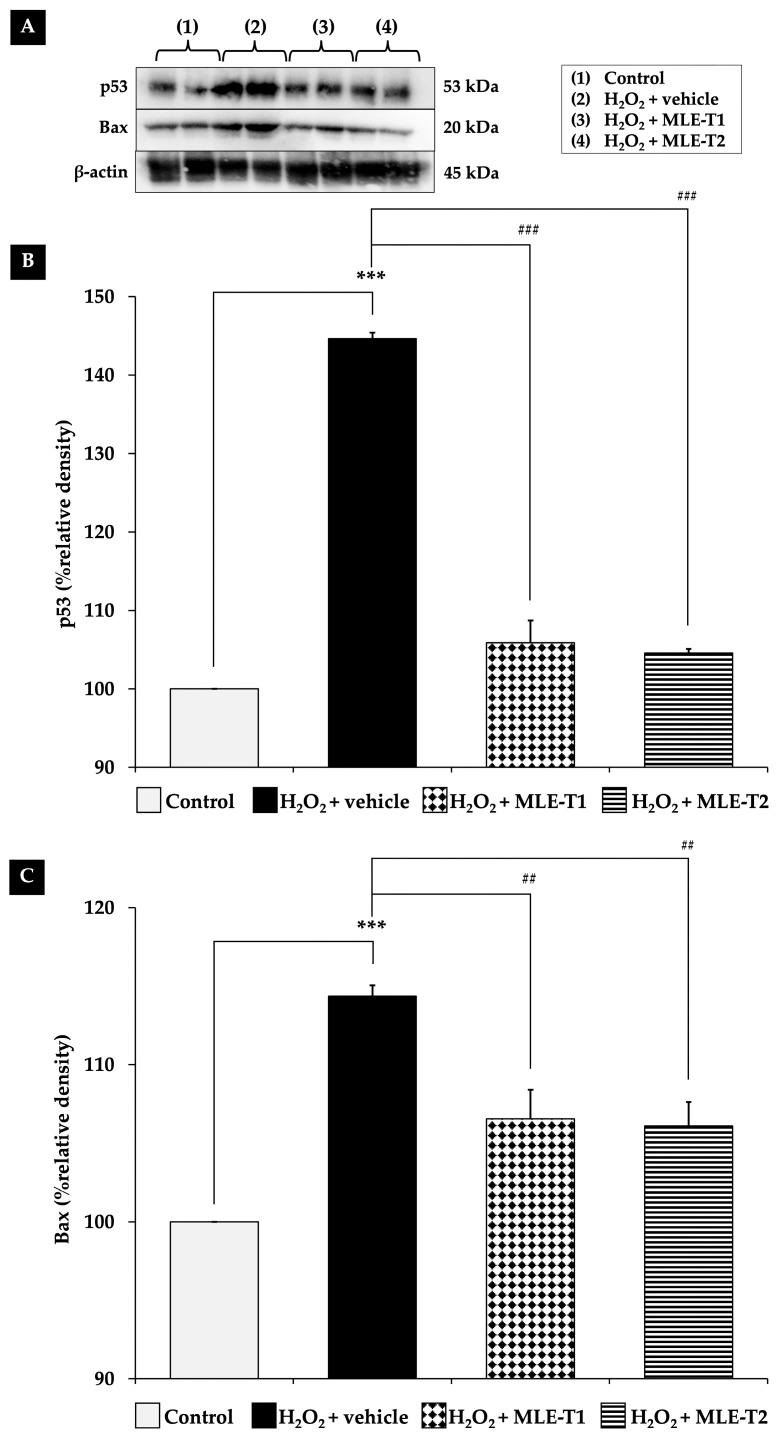
Effects of the selected mulberry leaf extract and Trolox combinations on p53 and Bax expression under H_2_O_2_-induced oxidative stress. (**A**) Representative Western blot images. (**B**) Quantitative analysis of p53 expression relative to the control. (**C**) Quantitative analysis of Bax expression relative to the control. Data are presented as mean ± SEM. *** *p* < 0.001 vs. control; ^##^ *p* < 0.05 and ^###^ *p* < 0.001 vs. H_2_O_2_ plus vehicle. H_2_O_2_, hydrogen peroxide; MLE-T1, mulberry leaf extract 1X plus Trolox 0.5X; MLE-T2, mulberry leaf extract 1X plus Trolox 1X; p53, tumor protein p53; Bax, Bcl-2-associated X protein.

To further clarify the involvement of apoptotic signaling, the expression of p53 and Bax proteins was examined. As shown in Figure 11, exposure to H_2_O_2_ plus vehicle markedly increased both p53 and Bax levels compared with the control group (*p* < 0.001 for all), indicating activation of pro-apoptotic pathways under oxidative stress. Treatment with the selected combinations significantly attenuated these elevations. Both MLE-T1 and MLE-T2 strongly reduced p53 expression (*p* < 0.001 vs. H_2_O_2_ plus vehicle), while Bax expression was moderately but significantly decreased (*p* < 0.01 vs. H_2_O_2_ plus vehicle).

### 3.11. Effects of Combined Treatment on the Bcl-2/Bax Ratio

**Figure 12:**
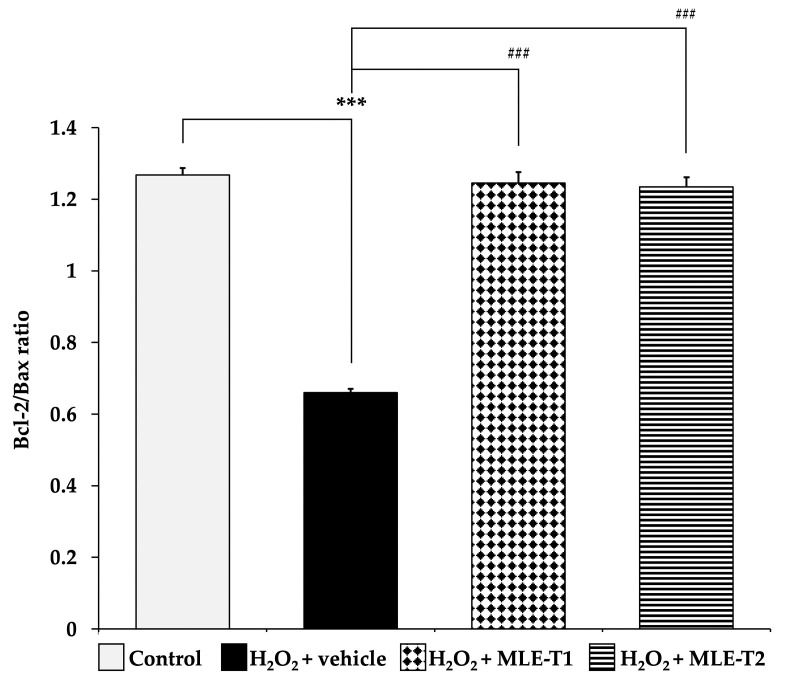
Effects of the selected mulberry leaf extract and Trolox combinations on the Bcl-2/Bax ratio under H_2_O_2_-induced oxidative stress. The Bcl-2/Bax ratio was calculated from the relative expression levels of Bcl-2 and Bax and is presented as mean ± SEM. *** *p* < 0.001 vs. control; ^###^ *p* < 0.001 vs. H_2_O_2_ plus vehicle. H_2_O_2_, hydrogen peroxide; MLE-T1, mulberry leaf extract 1X plus Trolox 0.5X; MLE-T2, mulberry leaf extract 1X plus Trolox 1X; Bcl-2, B-cell lymphoma 2; Bax, Bcl-2-associated X protein.

To further assess the overall balance between pro-survival and pro-apoptotic signaling, the Bcl-2/Bax ratio was calculated. As shown in Figure 12, H_2_O_2_ exposure markedly reduced the Bcl-2/Bax ratio compared with the control group (*p* < 0.001), indicating a shift toward apoptosis under oxidative stress conditions. In contrast, treatment with both MLE-T1 and MLE-T2 significantly restored the Bcl-2/Bax ratio (*p* < 0.001 vs. H_2_O_2_ plus vehicle).

## 4. Discussion

In the present study, we demonstrated that the combination of mulberry leaf extract and Trolox effectively protects SH-SY5Y neuroblastoma cells against H_2_O_2_-induced oxidative injury. This neuroprotective efficacy is achieved through a coordinated mechanism involving the upregulation of SIRT1 expression, resulting in the strengthening of intrinsic antioxidant defenses together with the modulation of downstream apoptosis-related signaling cascades.

To elucidate the specific bioactive components responsible for the observed neuroprotective effects, we characterized the chemical profile of the extract. Chromatographic analysis revealed that mulberry leaf extract contains chlorogenic acid, caffeic acid, rutin, naringin, and quercetin as major phenolic constituents. These compounds have been widely reported to exert antioxidant and cytoprotective effects through ROS scavenging, inhibition of lipid peroxidation, enhancement of endogenous antioxidant enzyme activity, modulation of redox-sensitive signaling pathways, and regulation of apoptosis-related proteins, including the Bcl-2 family [27,28,29,30,31,32].

However, despite these beneficial effects, the bioavailability and stability of mulberry leaf polyphenols under physiological conditions are limited by rapid metabolism, poor chemical stability, and low bioavailability, which may necessitate relatively high doses to achieve comparable efficacy in vivo [33,34]. In this context, combining mulberry leaf extract with Trolox offers a practical strategy to overcome these limitations, allowing effective biological responses at lower doses of each compound through complementary antioxidant and regulatory actions. Consistent with this, our findings demonstrated that the interaction between mulberry leaf extract and Trolox is model- and concentration-dependent. HSA and Loewe analyses indicated synergistic effects across multiple concentration pairs, particularly at moderate to higher doses, whereas ZIP and Bliss models suggested mainly additive or weak interactions at certain concentrations. These differences reflect the distinct assumptions of each model, with HSA and Loewe being more appropriate for agents with overlapping or related mechanisms, while ZIP and Bliss assume independent actions. The combined modulation of oxidative stress and apoptosis by both mulberry phytochemicals and Trolox [35,36,37] supports partial mechanistic overlap, consistent with stronger agreement in HSA and Loewe models. This interpretation was further supported by CI values ranging from 0.21 to 0.28 at optimal ratios, indicating synergistic interactions.

At the cellular level, exposure to H_2_O_2_ induced typical features of oxidative injury, including increased lipid peroxidation, elevated ROS levels, and suppression of endogenous antioxidant enzyme activities, resulting in reduced cell viability. Treatment with the selected combinations significantly improved cell survival compared with the H_2_O_2_-treated group. Mechanistically, these effects are closely associated with SIRT1 expression, which was markedly downregulated by H_2_O_2_ exposure but restored following treatment. Both mulberry leaf-derived bioactive compounds and Trolox have been reported to modulate redox balance and SIRT1-related signaling pathways. In particular, mulberry flavonoids have been linked to activation of the AMPK/SIRT1/PGC-1α axis [38], whereas Trolox has been shown to regulate redox homeostasis and cellular metabolism associated with SIRT1-related pathways [39]. Thus, SIRT1 upregulation may represent a key upstream regulatory mechanism mediating the observed cytoprotective effects.

Downstream of SIRT1, the protective effects may be explained through antioxidant and anti-apoptotic mechanisms. In the oxidative stress-related pathway, SIRT1 has been reported to enhance antioxidant defense through deacetylation of FOXO3a transcription factors, thereby promoting the transcription of endogenous antioxidant enzymes and improving cellular adaptation to oxidative stress [40]. In addition, SIRT1-dependent regulation of PGC-1α has been implicated in the maintenance of mitochondrial function and suppression of excessive ROS production [41]. These reported mechanisms are consistent with the findings of the present study, in which treatment with the combination reduced oxidative stress, as evidenced by decreased MDA and ROS levels, together with restoration of CAT, SOD, and GSH-Px activities, indicating reinforcement of endogenous antioxidant defenses and improved redox balance. However, FOXO3a and PGC-1α were not directly assessed in this study.

In the apoptotic pathway, SIRT1 has been reported to regulate mitochondrial-dependent apoptosis through multiple mechanisms, including modulation of the p53 signaling axis, where SIRT1-mediated deacetylation of p53 can suppress the expression of pro-apoptotic proteins such as Bax. In addition, SIRT1 has been shown to support pro-survival signaling through CREB activation, which contributes to the upregulation of anti-apoptotic proteins, including Bcl-2 [42,43]. Consistent with these reported mechanisms, the present study showed that H_2_O_2_ exposure increased p53 and Bax expression while decreasing CREB and Bcl-2 levels, indicating activation of intrinsic apoptotic signaling. Treatment with the combination reversed these alterations, suggesting suppression of pro-apoptotic signaling and enhancement of pro-survival pathways. The recovery of the Bcl-2/Bax ratio further indicates improved mitochondrial stability and reduced susceptibility to apoptosis. However, since these ratios were derived from densitometric analysis, they reflect relative protein expression changes rather than direct functional evidence of apoptotic execution.

**Figure 13:**
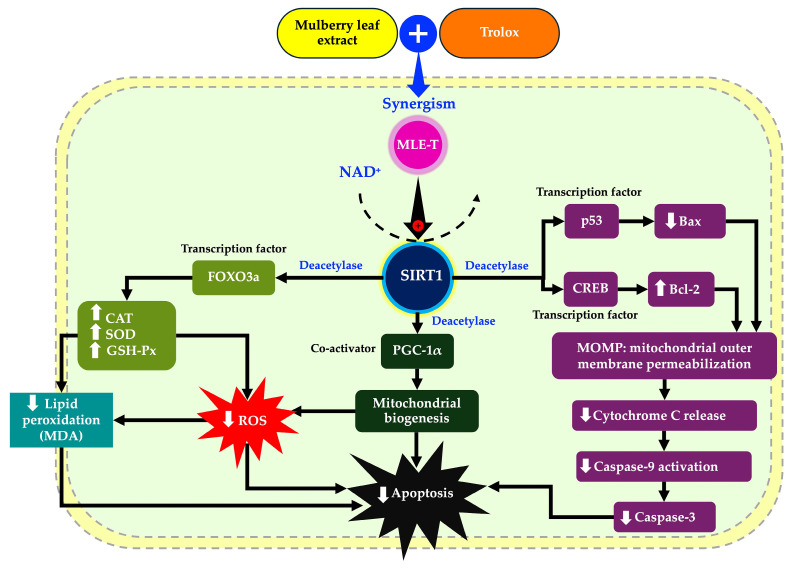
Proposed schematic diagram illustrating the potential neuroprotective pathways of mulberry leaf extract combined with Trolox against H_2_O_2_-induced oxidative stress in SH-SY5Y cells. The combination helps restore SIRT1 protein expression, which correlates with improved antioxidant enzyme defenses, decreased ROS and lipid peroxidation, and favorable modulation of apoptosis-related proteins (p53, Bax, CREB, and Bcl-2). SIRT1, sirtuin 1; ROS, reactive oxygen species; MDA, malondialdehyde; CAT, catalase; SOD, superoxide dismutase; GSH-Px, glutathione peroxidase; CREB, cyclic AMP response element-binding protein; Bcl-2, B-cell lymphoma 2; p53, tumor protein p53; Bax, Bcl-2-associated X protein.

In summary, our results suggest that the combination of mulberry leaf extract and Trolox protects SH-SY5Y cells against H_2_O_2_-induced oxidative stress through multiple coordinated mechanisms rather than a single antioxidant action, as illustrated in the proposed schematic mechanism (Figure 13). Beyond directly reducing ROS, the treatment increased SIRT1 expression, resulting in the restoration of endogenous antioxidant enzyme activities and the regulation of apoptosis-related proteins, thereby improving cell survival.

Despite these findings, several limitations of this study should be acknowledged. From a mechanistic perspective, not all downstream signaling events were fully characterized, and direct assessment of SIRT1-related epigenetic regulation was not performed, which limits definitive conclusions regarding upstream regulatory mechanisms. In addition, mitochondrial bioenergetic parameters including membrane potential and ATP production were not evaluated, and mitochondrial functional outcomes therefore remain to be clarified. From an experimental design perspective, the absence of single-agent treatment groups across mechanistic endpoints limits direct comparison and does not allow clear confirmation of synergistic versus additive effects at the molecular level, despite synergy analysis conducted in the cell viability experiments. Moreover, the live and dead assay was qualitative, which limits quantitative interpretation of cellular injury. From a cellular model perspective, the use of the cancer-derived SH-SY5Y cell line may not fully recapitulate physiological neuronal conditions. Although widely used in neurotoxicity studies, its transformed and undifferentiated state may influence cellular responses to oxidative stress and bioactive compounds. Finally, from a translational perspective, this in vitro experimental framework limits direct extrapolation to clinical application, and key factors such as bioavailability, metabolic stability, pharmacokinetics, and blood–brain barrier penetration require further in vivo and clinical investigation before practical application can be proposed.

## 5. Conclusions

In conclusion, the present study demonstrates that the combination of mulberry leaf extract and Trolox effectively protects SH-SY5Y cells against H_2_O_2_-induced oxidative injury. The observed neuroprotective effects were associated with regulation of SIRT1-associated oxidative stress and apoptotic signaling pathways. In addition, interaction analyses revealed a cooperative effect between mulberry leaf extract and Trolox, supporting the feasibility of this combinational approach. These findings provide preliminary evidence supporting the potential of mulberry leaf extract combined with Trolox as a functional food-based strategy to mitigate oxidative stress-related neuronal damage. However, because the present findings were obtained from an in vitro SH-SY5Y cell model, further investigations using differentiated neuronal cells, in vivo models, and clinical studies, together with exploration of additional molecular mechanisms, are warranted to address translational challenges and validate its long-term nutraceutical potential.

## Figures and Tables

**Table 1:** Validation results of the developed HPLC method for major constituents in mulberry leaf extract.

Parameters		Standard Compounds				
		Chlorogenic Acid	Caffeic Acid	Rutin	Naringin	Quercetin
LOD	Concentration (µg/mL)	0.1	0.1	0.1	0.1	0.25
	S/N	2.99 ± 0.05	3.30 ± 0.39	3.00 ± 0.06	2.99 ± 0.07	3.08 ± 0.02
LOQ	Concentration (µg/mL)	0.25	0.5	0.25	0.25	1.0
	S/N	10.12 ± 0.21	10.97 ± 0.65	10.25 ± 0.15	9.99 ± 0.10	10.00 ± 0.04
Linearity	Range (µg/mL)	0.25–10	0.1–5	0.25–10	0.25–10	0.25–10
	Equation	y = 17.802x − 5.2139	y = 58.349x − 5.4307	y = 98.345x − 30.123	y = 85.035x − 56.901	y = 10.178x − 4.2611
	(R^2^)	0.997	0.9943	0.9969	0.999	0.994
Precision (%RSD)	Repeatability	0.08–2.1%	1.42–2.93%	0.029–1.44%	0.24–1.89%	0.69–2.35%
	Intermediate precision	0.13–2.30%	0.16–2.28%	0.3–2.10%	0.05–2.60%	0.13–2.72%
Accuracy (%Recovery)	Low concentration	100.43 ± 1.89	101.11 ± 3.98	100.06 ± 2.76	100.20 ± 1.90	98.76 ± 1.02
	Medium concentration	99.80 ± 2.26	99.08 ± 1.62	101.23 ± 1.37	101.53 ± 1.106	98.90 ± 1.47
	High concentration	100.73 ± 1.76	97.71 ± 1.45	100.86 ± 1.00	100.9 ± 1.87	99.00 ± 3.20
Mulberry leaf extract (mg/g extract)		1.00 ± 0.01	0.12 ± 0.002	0.45 ± 0.001	0.36 ± 0.001	0.39 ± 0.01

Data are presented as mean ± SD. LOD, limit of detection; LOQ, limit of quantification; S/N, signal-to-noise ratio; R^2^, coefficient of determination; %RSD, percent relative standard deviation.

**Table 2:** Combination index values of mulberry leaf extract and Trolox.

Ratios (Dose Level)		%Cell Viability	Combination Index Value	Interaction
Mulberry Leaf Extract	Trolox			
0.125X	0X	48.85 ± 1.10	-	-
0.25X	0X	49.40 ± 0.08	-	-
0.5X	0X	50.96 ± 0.66	-	-
1X	0X	56.64 ± 0.98	-	-
2X	0X	53.38 ± 0.35	-	-
0.125X	0.125X	50.63 ± 0.67	1.85 ± 0.02	Antagonism
0.25X	0.125X	62.74 ± 0.45	1.41 ± 0.02	Moderate antagonism
0.5X	0.125X	68.54 ± 0.15	1.36 ± 0.01	Moderate antagonism
1X	0.125X	80.23 ± 0.84	1.16 ± 0.06	Slight antagonism
2X	0.125X	70.33 ± 1.18	1.51 ± 0.06	Antagonism
0.125X	0.25X	65.30 ± 0.69	1.30 ± 0.03	Moderate antagonism
0.25X	0.25X	66.69 ± 0.46	1.26 ± 0.02	Moderate antagonism
0.5X	0.25X	78.89 ± 1.16	0.91 ± 0.05	Nearly additive effect
1X	0.25X	88.28 ± 0.64	0.69 ± 0.03	Synergism
2X	0.25X	77.26 ± 1.37	1.15 ± 0.07	Slight antagonism
0.125X	0.5X	79.68 ± 0.40	0.76 ± 0.02	Moderate synergism
0.25X	0.5X	81.96 ± 1.17	0.68 ± 0.04	Synergism
0.5X	0.5X	88.96 ± 0.58	0.48 ± 0.02	Synergism
1X	0.5X	95.14 ± 0.57	0.28 ± 0.03	Strong synergism
2X	0.5X	74.73 ± 0.49	1.28 ± 0.03	Moderate antagonism
0.125X	1X	77.96 ± 0.58	0.83 ± 0.03	Moderate synergism
0.25X	1X	83.76 ± 1.44	0.62 ± 0.05	Synergism
0.5X	1X	91.79 ± 0.63	0.35 ± 0.03	Synergism
1X	1X	96.36 ± 0.25	0.21 ± 0.02	Strong synergism
2X	1X	78.06 ± 0.60	1.11 ± 0.04	Slight antagonism
0.125X	2X	68.40 ± 0.39	1.18 ± 0.02	Slight antagonism
0.25X	2X	70.03 ± 0.73	1.14 ± 0.03	Slight antagonism
0.5X	2X	71.10 ± 0.98	1.24 ± 0.04	Moderate antagonism
1X	2X	74.13 ± 0.76	1.52 ± 0.05	Antagonism
2X	2X	66.00 ± 0.53	1.73 ± 0.04	Antagonism
0X	0.125X	44.11 ± 0.49	-	-
0X	0.25X	44.91 ± 0.33	-	-
0X	0.5X	55.90 ± 0.31	-	-
0X	1X	71.86 ± 0.48	-	-
0X	2X	66.44 ± 0.63	-	-

Data are presented as mean ± SEM (n = 3).

## Data Availability

All data generated or analyzed in this study are included in the article and its Supplementary Materials. Further information can be obtained from the corresponding author upon request.

## Supplementary Materials

The following supporting information can be downloaded at: https://www.mdpi.com/article/10.3390/foods15111974/s1. Figure S1: Original images of SIRT1 expression. Figure S2: Original images of CREB expression. Figure S3: Original images of Bcl-2 expression. Figure S4: Original images of p53 expression. Figure S5: Original images of Bax expression. Figures S6–S8: Original images of β-actin expression. Figure S9: Original microscopic images of SH-SY5Y cell morphology. Table S1: Original input data for dose–response and synergy analysis of mulberry leaf extract and Trolox.
