# Supplementary material for: A Potential Functional Food-Based Neuroprotective Strategy Using Mulberry Leaf Extract and Trolox Against H2O2-Induced Oxidative Stress in SH-SY5Y Cells
Source: Foods. 2026 Jun 2;15(11):1974. doi: 10.3390/foods15111974 (PMC13256257; doi:10.3390/foods15111974)
Supplement: Supplementary file 1 [file foods-15-01974-s001.zip › foods-4337352-supplementary.pdf]

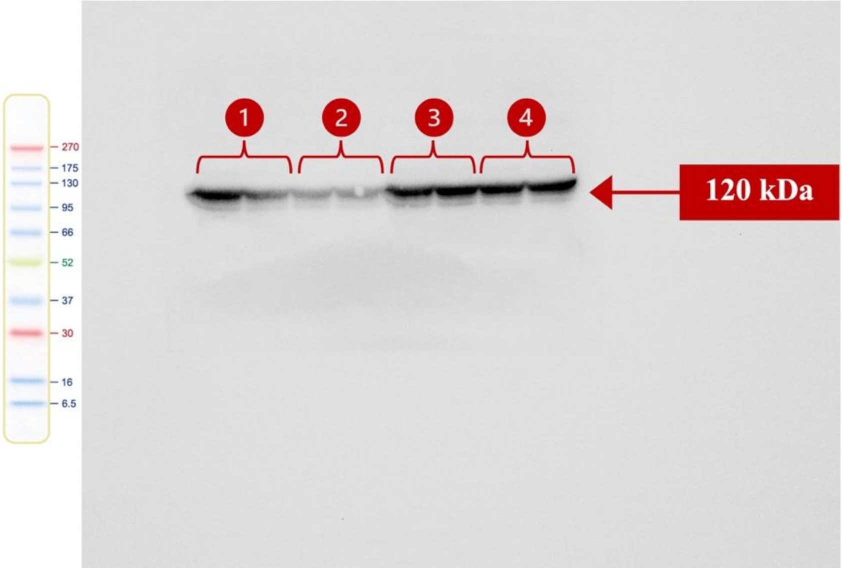

Figure S1: Western blot analysis of SIRT1 expression in SH-SY5Y cells following treatment with the selected mulberry leaf extract and Trolox combinations against H_2O_2-induced neurotoxicity. The experimental groups were: (1) control, (2) H_2O_2 + vehicle, (3) H_2O_2 + MEL-T1, and (4) H_2O_2 + MEL-T2. MLE-T1, mulberry leaf extract 1X plus Trolox 0.5X; MLE-T2, mulberry leaf extract 1X plus Trolox 1X. This figure shows the original, unprocessed, uncropped, full-length membrane images.

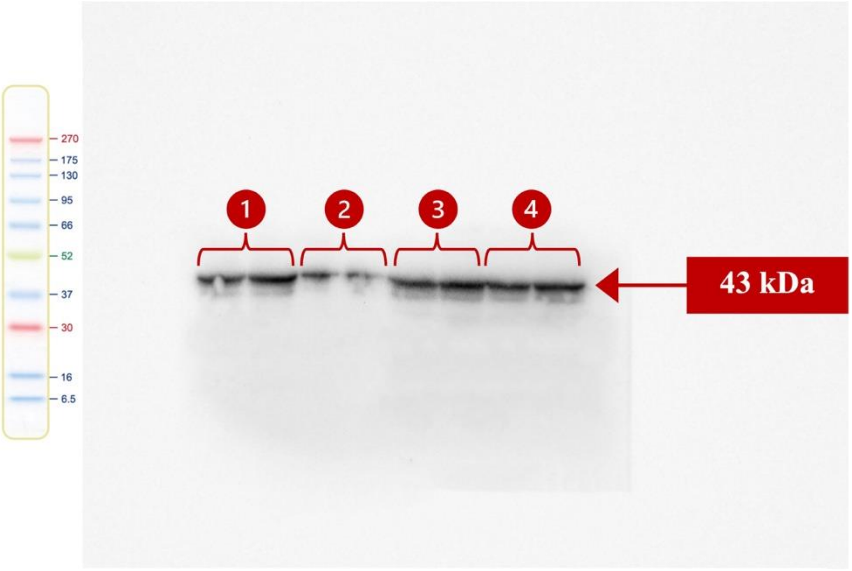

Figure S2: Western blot analysis of CREB expression in SH-SY5Y cells following treatment with the selected mulberry leaf extract and Trolox combinations against H_2O_2-induced neurotoxicity. The experimental groups were: (1) control, (2) H_2O_2 + vehicle, (3) H_2O_2 + MEL-T1, and (4) H_2O_2 + MEL-T2. MLE-T1, mulberry leaf extract 1X plus Trolox 0.5X; MLE-T2, mulberry leaf extract 1X plus Trolox 1X. This figure shows the original, unprocessed, uncropped, full-length membrane images.

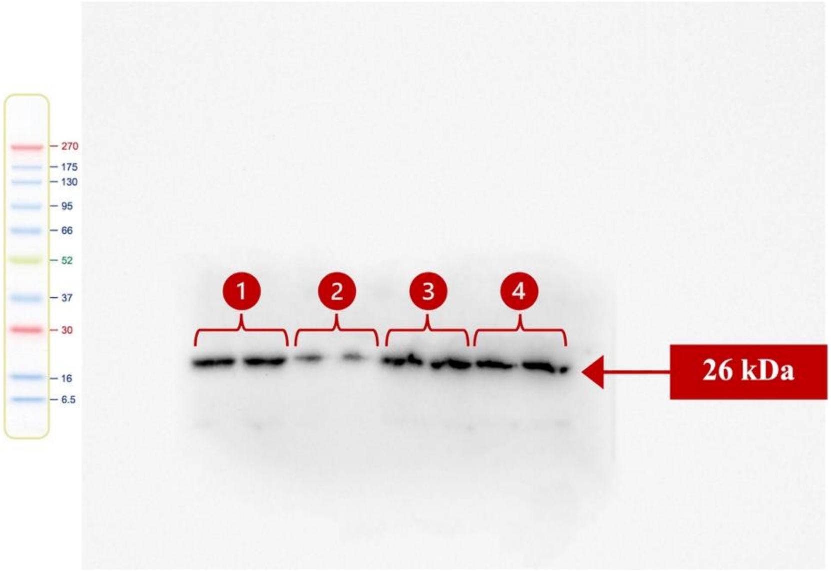

Figure S3: Western blot analysis of Bcl-2 expression in SH-SY5Y cells following treatment with the selected mulberry leaf extract and Trolox combinations against H_2O_2-induced neurotoxicity. The experimental groups were: (1) control, (2) H_2O_2 + vehicle, (3) H_2O_2 + MEL-T1, and (4) H_2O_2 + MEL-T2. MLE-T1, mulberry leaf extract 1X plus Trolox 0.5X; MLE-T2, mulberry leaf extract 1X plus Trolox 1X. This figure shows the original, unprocessed, uncropped, full-length membrane images.

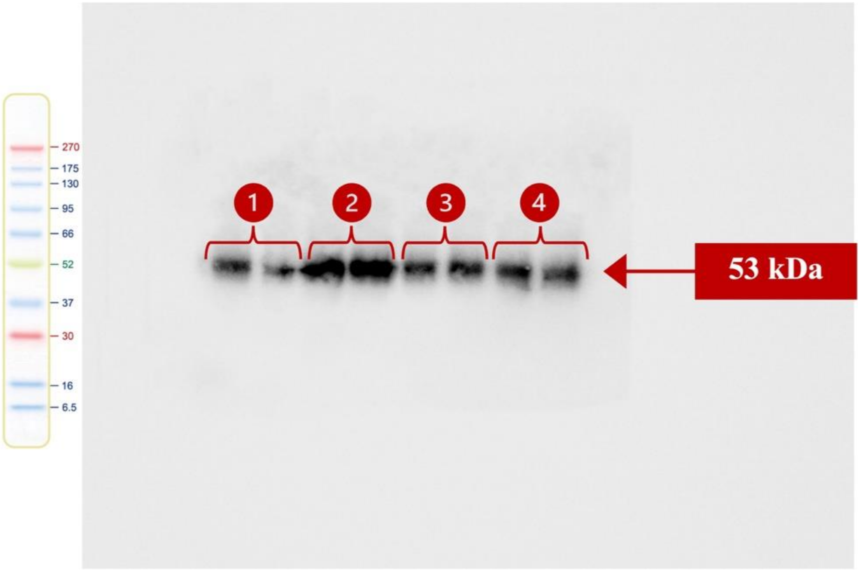

Figure S4: Western blot analysis of p53 expression in SH-SY5Y cells following treatment with the selected mulberry leaf extract and Trolox combinations against H_2O_2-induced neurotoxicity. The experimental groups were: (1) control, (2) H_2O_2 + vehicle, (3) H_2O_2 + MEL-T1, and (4) H_2O_2 + MEL-T2. MLE-T1, mulberry leaf extract 1X plus Trolox 0.5X; MLE-T2, mulberry leaf extract 1X plus Trolox 1X. This figure shows the original, unprocessed, uncropped, full-length membrane images.

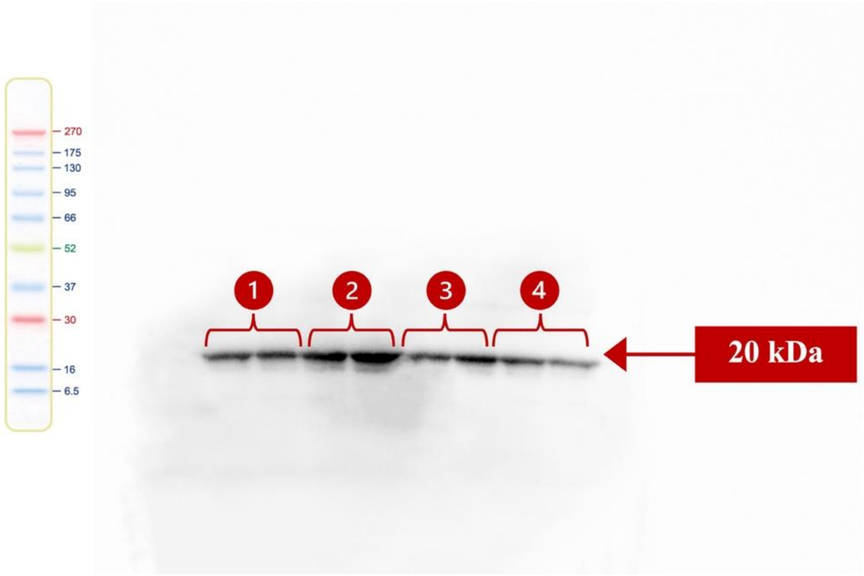

Figure S5: Western blot analysis of Bax expression in SH-SY5Y cells following treatment with the selected mulberry leaf extract and Trolox combinations against H_2O_2-induced neurotoxicity. The experimental groups were: (1) control, (2) H_2O_2 + vehicle, (3) H_2O_2 + MEL-T1, and (4) H_2O_2 + MEL-T2. MLE-T1, mulberry leaf extract 1X plus Trolox 0.5X; MLE-T2, mulberry leaf extract 1X plus Trolox 1X. This figure shows the original, unprocessed, uncropped, full-length membrane images.

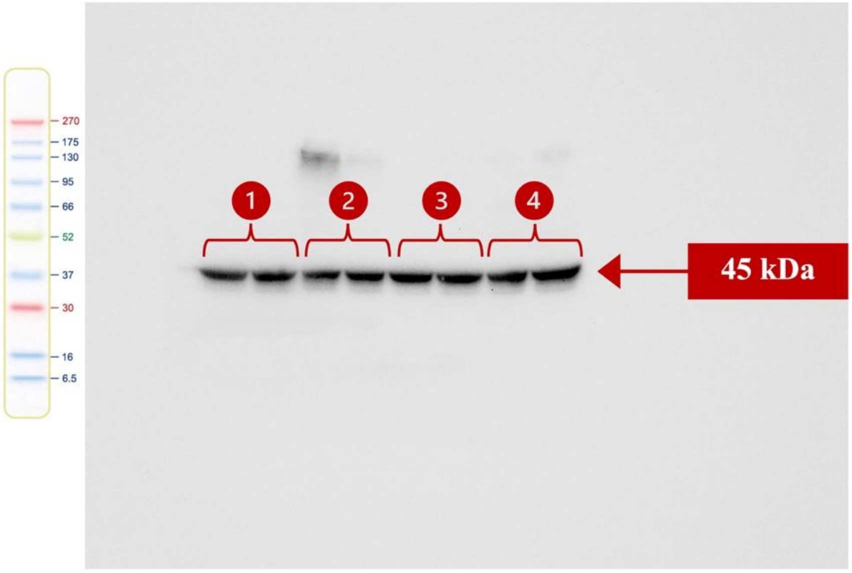

Figure S6: Western blot analysis of bata-actin expression in SH-SY5Y cells following treatment with the selected mulberry leaf extract and Trolox combinations against H_2O_2-induced neurotoxicity. The experimental groups were: (1) control, (2) H_2O_2 + vehicle, (3) H_2O_2 + MEL-T1, and (4) H_2O_2 + MEL-T2. MLE-T1, mulberry leaf extract 1X plus Trolox 0.5X; MLE-T2, mulberry leaf extract 1X plus Trolox 1X. This figure shows the original, unprocessed, uncropped, full-length membrane images.

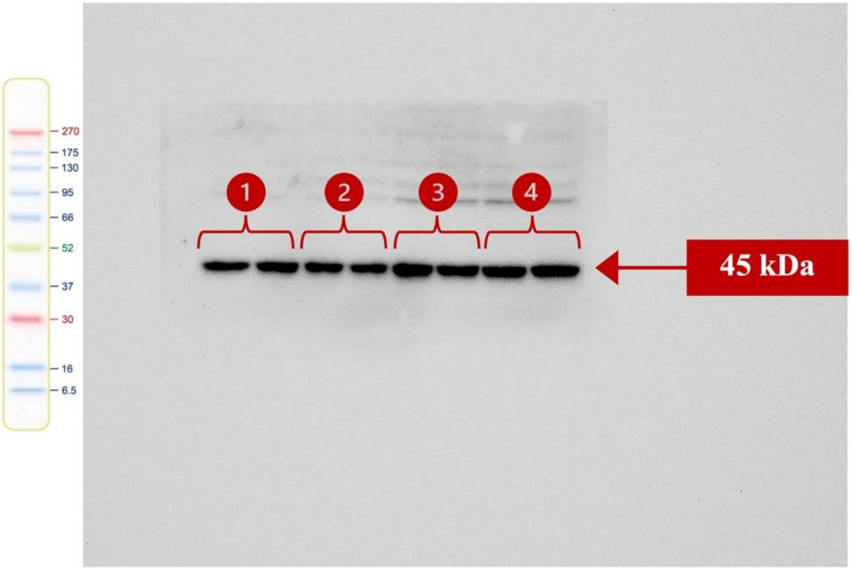

Figure S7: Western blot analysis of bata-actin expression in SH-SY5Y cells following treatment with the selected mulberry leaf extract and Trolox combinations against H_2O_2-induced neurotoxicity. The experimental groups were: (1) control, (2) H_2O_2 + vehicle, (3) H_2O_2 + MEL-T1, and (4) H_2O_2 + MEL-T2. MLE-T1, mulberry leaf extract 1X plus Trolox 0.5X; MLE-T2, mulberry leaf extract 1X plus Trolox 1X. This figure shows the original, unprocessed, uncropped, full-length membrane images.

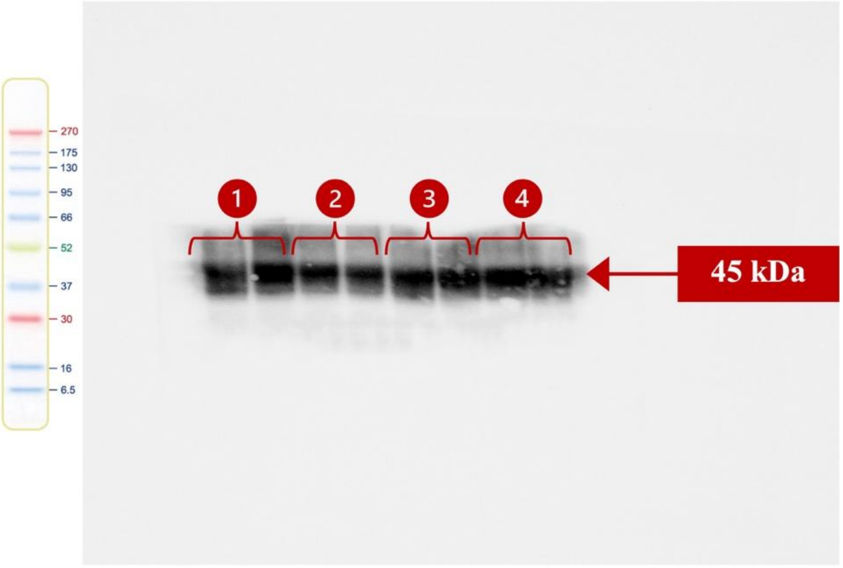

Figure S8: Western blot analysis of bata-actin expression in SH-SY5Y cells following treatment with the selected mulberry leaf extract and Trolox combinations against H_2O_2-induced neurotoxicity. The experimental groups were: (1) control, (2) H_2O_2 + vehicle, (3) H_2O_2 + MEL-T1, and (4) H_2O_2 + MEL-T2. MLE-T1, mulberry leaf extract 1X plus Trolox 0.5X; MLE-T2, mulberry leaf extract 1X plus Trolox 1X. This figure shows the original, unprocessed, uncropped, full-length membrane images.

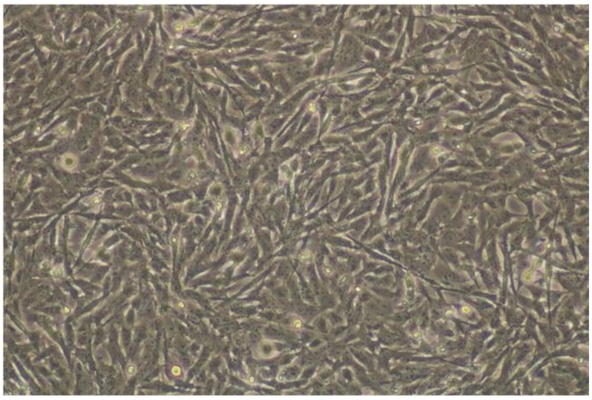

(1)

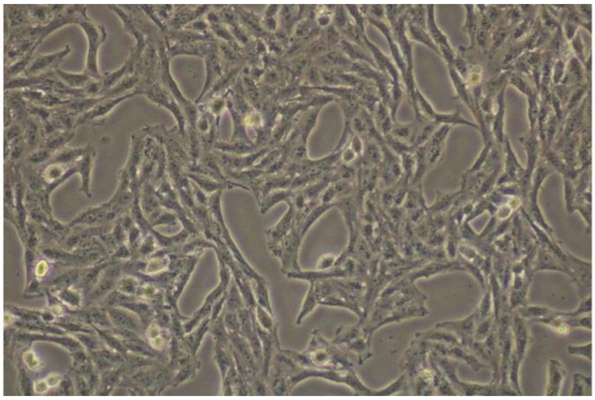

(2)

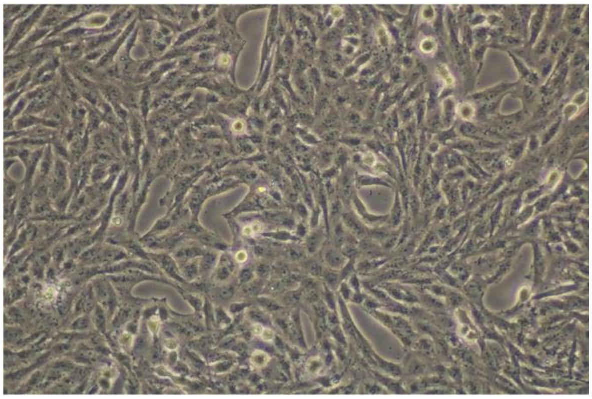

(3)

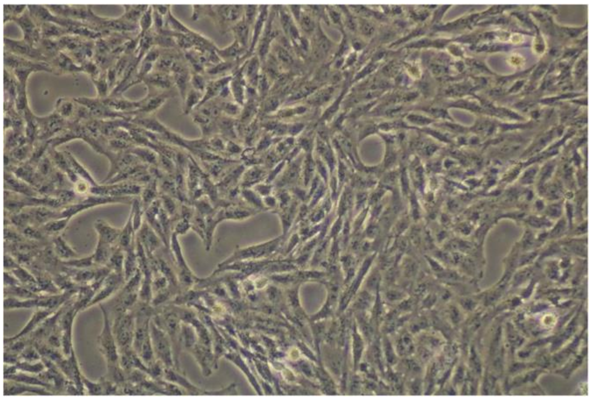

(4)

Figure S9: Original microscopic images of SH-SY5Y cell morphology captured at 10× magnification following treatment with the selected combinations of mulberry leaf extract and Trolox against H_2O_2-induced neurotoxicity. The experimental groups were: (1) control, (2) H_2O_2 + vehicle, (3) H_2O_2 + MEL-T1, and (4) H_2O_2 + MEL-T2. MLE-T1, mulberry leaf extract 1X plus Trolox 0.5X; MLE-T2, mulberry leaf extract 1X plus Trolox 1X.

Table S1. Original input data for dose–response and synergy analysis of mulberry leaf extract and Trolox

No.	Mulberry leaf extract (MLE)	Trolox	PairIndex	Drug 1	Drug 2	Conc1	Conc2	Viability (%)	Inhibition (%)
1	0.125X	0X	1	MLF (ug/mL)	Trolox (uM)	3.75	0.00	48.85	51.15
2	0.25X	0X	1	MLF (ug/mL)	Trolox (uM)	7.50	0.00	49.40	50.6
3	0.5X	0X	1	MLF (ug/mL)	Trolox (uM)	15.00	0.00	50.96	49.0375
4	1X	0X	1	MLF (ug/mL)	Trolox (uM)	30.00	0.00	56.64	43.3625
5	2X	0X	1	MLF (ug/mL)	Trolox (uM)	60.00	0.00	52.38	47.625
6	0.125X	0.125X	1	MLF (ug/mL)	Trolox (uM)	3.75	2.25	50.63	49.375
7	0.25X	0.125X	1	MLF (ug/mL)	Trolox (uM)	7.50	2.25	62.74	37.2625
8	0.5X	0.125X	1	MLF (ug/mL)	Trolox (uM)	15.00	2.25	68.54	31.4625
9	1X	0.125X	1	MLF (ug/mL)	Trolox (uM)	30.00	2.25	80.23	19.775
10	2X	0.125X	1	MLF (ug/mL)	Trolox (uM)	60.00	2.25	70.33	29.675
11	0.125X	0.25X	1	MLF (ug/mL)	Trolox (uM)	3.75	4.50	65.30	34.7
12	0.25X	0.25X	1	MLF (ug/mL)	Trolox (uM)	7.50	4.50	66.69	33.3125
13	0.5X	0.25X	1	MLF (ug/mL)	Trolox (uM)	15.00	4.50	78.89	21.1125
14	1X	0.25X	1	MLF (ug/mL)	Trolox (uM)	30.00	4.50	88.28	11.725
15	2X	0.25X	1	MLF (ug/mL)	Trolox (uM)	60.00	4.50	77.26	22.7375
16	0.125X	0.5X	1	MLF (ug/mL)	Trolox (uM)	3.75	9.00	79.68	20.325
17	0.25X	0.5X	1	MLF (ug/mL)	Trolox (uM)	7.50	9.00	81.96	18.0375
18	0.5X	0.5X	1	MLF (ug/mL)	Trolox (uM)	15.00	9.00	88.96	11.0375
19	1X	0.5X	1	MLF (ug/mL)	Trolox (uM)	30.00	9.00	95.14	4.8625
20	2X	0.5X	1	MLF (ug/mL)	Trolox (uM)	60.00	9.00	74.73	25.275
21	0.125X	1X	1	MLF (ug/mL)	Trolox (uM)	3.75	18.00	77.96	22.0375
22	0.25X	1X	1	MLF (ug/mL)	Trolox (uM)	7.50	18.00	83.76	16.2375
23	0.5X	1X	1	MLF (ug/mL)	Trolox (uM)	15.00	18.00	91.79	8.2125
24	1X	1X	1	MLF (ug/mL)	Trolox (uM)	30.00	18.00	96.36	3.6375
25	2X	1X	1	MLF (ug/mL)	Trolox (uM)	60.00	18.00	78.06	21.9375
26	0.125X	2X	1	MLF (ug/mL)	Trolox (uM)	3.75	36.00	68.40	31.6
27	0.25X	2X	1	MLF (ug/mL)	Trolox (uM)	7.50	36.00	70.03	29.975
28	0.5X	2X	1	MLF (ug/mL)	Trolox (uM)	15.00	36.00	71.10	28.9
29	1X	2X	1	MLF (ug/mL)	Trolox (uM)	30.00	36.00	74.13	25.875
30	2X	2X	1	MLF (ug/mL)	Trolox (uM)	60.00	36.00	66.00	34
31	0X	0.125X	1	MLF (ug/mL)	Trolox (uM)	0.00	2.25	44.11	55.8875
32	0X	0.25X	1	MLF (ug/mL)	Trolox (uM)	0.00	4.50	44.91	55.0875
33	0X	0.5X	1	MLF (ug/mL)	Trolox (uM)	0.00	9.00	55.90	44.1
34	0X	1X	1	MLF (ug/mL)	Trolox (uM)	0.00	18.00	71.86	28.1375
35	0X	2X	1	MLF (ug/mL)	Trolox (uM)	0.00	36.00	66.44	33.5625
